# Neuroanatomical Evidence That Kisspeptin Directly Regulates Isotocin and Vasotocin Neurons

**DOI:** 10.1371/journal.pone.0062776

**Published:** 2013-04-25

**Authors:** Shinji Kanda, Yasuhisa Akazome, Yuta Mitani, Kataaki Okubo, Yoshitaka Oka

**Affiliations:** 1 Department of Biological Sciences, Graduate School of Science, The University of Tokyo, Tokyo, Japan; 2 Department of Aquatic Bioscience, Graduate School of Agricultural and Life Sciences, The University of Tokyo, Tokyo, Japan; University of Rouen, France

## Abstract

Neuropeptide kisspeptin has been suggested to be an essential central regulator of reproduction in response to changes in serum gonadal steroid concentrations. However, in spite of wide kisspeptin receptor distribution in the brain, especially in the preoptic area and hypothalamus, the research focus has mostly been confined to the kisspeptin regulation on GnRH neurons. Here, by using medaka whose kisspeptin (*kiss1*) neurons have been clearly demonstrated to be regulated by sex steroids, we analyzed the anatomical distribution of kisspeptin receptors Gpr54-1 and Gpr54-2. Because the both receptors were shown to be activated by kisspeptins (Kiss1 and Kiss2), we analyzed the anatomical distribution of the both receptors by *in situ* hybridization. They were mainly expressed in the ventral telencephalon, preoptic area, and hypothalamus, which have been suggested to be involved in homeostatic functions including reproduction. First, we found *gpr54-2* mRNA expression in nucleus preopticus pars magnocellularis and demonstrated that *vasotocin* and *isotocin* (*Vasopressin* and *Oxytocin* ortholog, respectively) neurons express *gpr54-2* by dual *in situ* hybridization. Given that kisspeptin administration increases serum oxytocin and vasopressin concentration in mammals, the present finding are likely to be vertebrate-wide phenomenon, although direct regulation has not yet been demonstrated in mammals. We then analyzed co-expression of kisspeptin receptors in three types of GnRH neurons. It was clearly demonstrated that *gpr54*-expressing cells were located adjacent to GnRH1 neurons, although they were not GnRH1 neurons themselves. In contrast, there was no *gpr54*-expressing cell in the vicinities of neuromodulatory GnRH2 or GnRH3 neurons. From these results, we suggest that medaka kisspeptin neurons directly regulate some behavioral and neuroendocrine functions via vasotocin/isotocin neurons, whereas they do not regulate hypophysiotropic GnRH1 neurons at least in a direct manner. Thus, direct kisspeptin regulation of GnRH1 neurons proposed in mammals may not be the universal feature of vertebrate kisspeptin system in general.

## Introduction

Recent studies of human hypogonadotropic hypogonadism and knockout mice for the kisspeptin gene *Kiss1* and the receptor gene *Gpr54* have revealed that the kisspeptin/GPR54 system is essential for the mammalian reproductive functions [1]–[5]. Subsequently, *in vitro* electrophysiological experiments using GnRH-GFP transgenic mice showed that kisspeptin has a persistent depolarizing effect on the GnRH neurons, which are supposed to be the basis for facilitation of release of GnRH [6]–[10]. Moreover, kisspeptin is also reported to facilitate gonadotropin release *in vivo* as well. Thus, kisspeptin system has been attracting much attention of neuroendocrinologists. However, limited number of studies have assessed the function of kisspeptin-GPR54 system in non-mammalian species. In addition, little is known about the physiological role(s) of kisspeptin-GPR54 system in non-reproductive functions even in mammals.

Therefore, for the basis of full understanding of the general physiological roles of kisspeptins in vertebrate brains, we examined distribution of kisspeptin receptor *gpr54-1* and *gpr54-2* in the brain of medaka. Medaka is a small teleost fish that is widely used as a model animal because of many advantages, such as availability of genome database and easy access to various genetic tools. Because of the lack of knowledge on kisspeptin functions in non-mammalian vertebrates, such a model animal should go a long way towards the understandings of both evolution and general functions of the kisspeptin systems in vertebrates. In the present study, our *gpr54* in situ hybridization study has shown that kisspeptin receptors are expressed by neurons in the preoptic area and hypothalamus, which are supposed to be involved in the regulation of homeostasis and instinctive behaviors. After careful preliminary experiments for identification of kisspeptin receptor expressing neurons, we demonstrated the co-expression of kisspeptin receptor mRNA in magnocellular vasotocin and isotocin neurons for the first time in vertebrates. Oxytocin (isotocin in teleosts) and vasopressin (vasotocin in teleosts) neurons are well-known to be involved in the regulation of reproduction-related and other behaviors in many vertebrate species(reviewed in [11], [12]). As in mammals, teleost isotocin and vasotocin neurons are suggested to be involved in the regulation of social behaviors including sex behaviors and aggressive behavior, although the effects vary among species [13] Therefore, together with the fact that medaka kisspeptin neurons increase their *kiss1* mRNA expression in the breeding state, kisspeptin may mediate information regarding the breeding state to the isotocin and vasotocin neurons, which leads to the state-dependent behavioral modulation. In addition to these novel findings, we examined the possible co-expression of kisspeptin receptors in three functionally distinct GnRH systems. Recent studies have shown that most vertebrates have one or two extra-hypothalamic GnRH neuronal systems, whose cell bodies are localized in the midbrain tegmentum (TEG-GnRH2 neurons) and the terminal nerve (TN-GnRH3 neurons) in addition to the conventional hypophysiotropic GnRH (GnRH1) system, and all three GnRH systems are well developed in teleosts [14], [15]. Our double in situ hybridization study showed anatomical evidence to suggest the lack of direct kisspeptin regulation and leaves the possibility of indirect regulation via neighboring interneurons on hypophysiotropic GnRH1 neurons, whereas kisspeptins do not have such regulation on neuromodulatory GnRH2 and 3 neurons.

## Materials and Methods

### Animals

Male and female d-rR strain medaka (*Oryzias latipes*; teleost fish) were maintained under a 14 h light/10 h dark photoperiod at a temperature of 27°C. The fish were fed twice daily with live brine shrimp and flake food. The animals were maintained and used in accordance with the guidelines of The Physiological Society of Japan and the University of Tokyo for the Use and Care of Experimental Animals. We used only anamniotes, which do not require any permission by the University of Tokyo for the Use and Care of Experimental Animals.

### Luciferase Assays

The decapeptide of medaka Kiss1 (Kiss1 (10): YNLNSFGLRY-NH_2_, believed to be the core peptide sequence for its physiological function), pentadecapeptide of Kiss1 (Kiss1 (15): pEDLSSYNLNSFGLRY-NH_2_) and dodecapeptide of medaka Kiss2 (Kiss2 (12): SKFNYNPFGLRF-NH_2_) were synthesized (Sigma-Aldrich Japan, Tokyo, Japan; Scrum, Tokyo, Japan; Bonac Corporation, Kurume, Japan, respectively).

The cDNA clones containing full-length open reading frames of *gpr54-1* and *gpr54-2* were subcloned into the expression vector pcDNA3.1 (Invitrogen). COS-7 cells were grown at 37°C in Dulbecco’s modified Eagle’s medium (DMEM), supplemented with 10% foetal bovine serum. One day before transfection, the cells were seeded into 24-well plates. The plasmid DNAs (100 ng/well) were transfected into monolayer culture cells with either pSRE-Luc or pCRE-Luc (100 ng/well; Clontech, Palo Alto, CA), and pRL-CMV containing the Renilla luciferase reporter gene (2.5 ng/well; Promega, Madison, WI), using Lipofectamine LTX (Invitrogen). The cells were maintained in a serum-free medium for 24 hours. After that, they were incubated with various concentrations (from 0 to 10^−5^ M) of medaka Kiss1 or Kiss2 for six hours and then harvested and analyzed. Luciferase activity in the cell extract was measured using Dual-Glo Luciferase Assay System (Promega) with Lumat LB9507 (EB & G Berthold, Bad Wildbad, Germany).

### In situ Hybridization

For the *in situ* hybridization analysis, we used sexually mature male and female medaka pairs that had oviposited fertilized eggs on the day of the fixation.

The medaka was deeply anesthetized with MS-222 (Sigma, St. Louis, MO, USA) and perfused with 4% paraformaldehyde in 0.05 M phosphate buffered saline (PBS) from the conus anteriosus. The brain was postfixed with the same fixative at least 1 h at 4°C. They were then embedded in 5% agarose (Sigma Type IX) solution containing 20% sucrose and were quickly frozen in n-hexane (∼−60°C). Complete serial frontal sections were cut on a cryostat at 20 µm, and dried at room temperature (RT) for at least 2 h. To detect mRNA, we prepared a gene-specific digoxigenin (DIG)-labeled probe and performed nonradioactive *in situ* hybridization based on the methods as previously reported [16]. The sections were hybridized with 100 ng/ml DIG-labelled antisense cRNA probes (*kiss1*, position 21-365, AB272755; *gpr54-1*, position 1–1101 of ENSORLT00000002103, chromosome 9 4480521-4500733; *gpr54-2*, position −6–1125 of ENSORLT00000022192, chromosome 17, 29854753-29839926; for *gnrh2*, *gnrh3*, *isotocin*, and *vasotocin*, probes were synthesized based on the previous report [17] ) synthesized from the medaka brain cDNA using a labelling kit (Roche Molecular Biochemicals GmbH, Mannheim, Germany) overnight at 58°C. A sense RNA probe was used as a negative control (nomenclature based on Lee et al.; *gpr54-1* corresponds to *kissrb* in [18] and *kissr2* in [19] whereas *gpr54-2* corresponds to *kiss1ra* in [18] and *kissr4* in [19]; see [20]).

The sections were observed under the light microscope. For the nomenclature of the medaka brain nuclei, we followed the Medaka Histological Atlas (Wakamatsu *et al*., Medaka Histological Atlas, edited by the Editorial Board of Medaka Histological Atlas of NBRP Medaka, http://www.shigen.nig.ac.jp/medaka/medaka_atlas) throughout this study.

### Dual in situ Hybridization for Isotocin/Vasotocin/gnrh and gpr54-1/gpr54-2

For the dual *in situ* hybridization study, we used a mixture of fluorescein-labelled *gnrh1* (position 1–428, NM001104699) or *vasotocin* (position, 1–807, AB691137) or *isotocin* (position 1–739, AB691138) probe (specificities were confirmed by the presence or absence of signals using the antisense or sense probes) and the DIG-labeled *gpr54-1/gpr54-2* probe (described above). Dual in situ hybridization signals were visualized as previously described [21] using an HNPP fluorescence detection kit (Roche) or Fast-Red substrate kit (abcam, Cambridge, UK) according to the manufacturer’s instruction. We used long day-conditioned (LD, 14 hour light 10 hour dark; breeding condition) and short day-conditioned (SD, 10 hour light 14 hour dark; non-breeding condition) medaka for the analysis of the percentages of colocalization. The fluorescence was observed under confocal laser-scanning microscope LSM-710 (Carl Zeiss, Oberkochen, Germany). For GnRH2 and 3 neurons, because in situ hybridization using adjacent sections proved that cells near GnRH2 or GnRH3 neurons did not express *gpr54-1* or *gpr54-2* mRNA, we did not perform the double labeling and showed adjacent sections instead?

## Results

### Both Kiss1 and Kiss2 Activate Both Subtypes of gpr54

**Figure 1:**
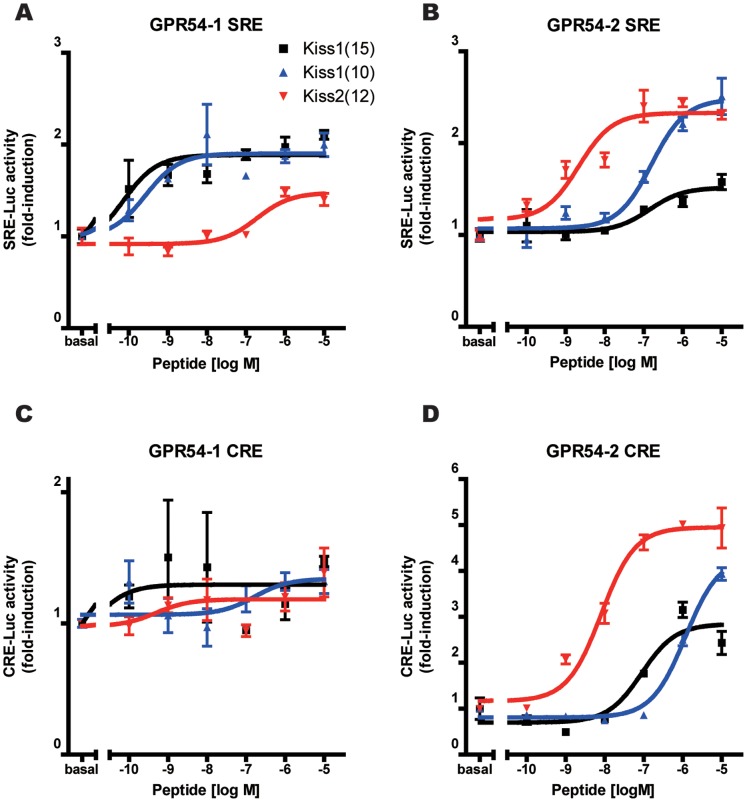
Luciferase assays for the activation of two types of receptors, Gpr54-1 and Gpr54-2, by the ligands, Kiss1 and Kiss2. **Medaka ***gpr54-1* (A, C) or *gpr54-2* (B, D) cDNA was transfected to COS-7 cells with SRE-luc or CRE-luc vector.**** Various concentrations of medaka Kiss1 and Kiss2 were applied to the culture medium, and the luciferase activity was measured. The results are indicated as mean ± SEM, each of which was conducted in triplicates. The data are expressed as the ratio of changes in luciferase activity over the control renilla luciferase activity.

To assess GPR54 stimulating activity of Kiss1 and Kiss2, we carried out a luciferase reporter assay for the two types of Gpr54 receptors in medaka, Gpr54-1 and Gpr54-2. The luciferase assay showed that both Kiss1 and Kiss2 significantly activate Gpr54-1 as well as Gpr54-2 (Fig. 1). To examine the signal transduction pathway for each type of medaka Gpr54, SRE- or CRE-driven luciferase reporter gene assay was performed using COS-7 cells. For SRE-luc reporter system, the post-receptor signaling pathway of Gpr54-2 was similarly and significantly activated by both Kiss1 and Kiss2 (Fig. 1B), whereas Gpr54-1 was only slightly activated by Kiss1 but not by Kiss2 (Fig. 1A). For CRE-luc reporter system, both Gpr54-1 and Gpr54-2 were activated by both peptides, although Kiss2 showed a higher potency than Kiss1 (Fig. 1C and 1D). In the present analysis, the CRE-driven luciferase activity in Gpr54-2 expressing cells showed the strongest dose-dependent response to Kiss1/Kiss2 application (Fig. 1D).

### Two Subtypes of Kisspeptin Receptors are Expressed Mainly in the Ventral Telencephalon, Preoptic Area, Habenula, and Hypothalamus

**Figure 2:**
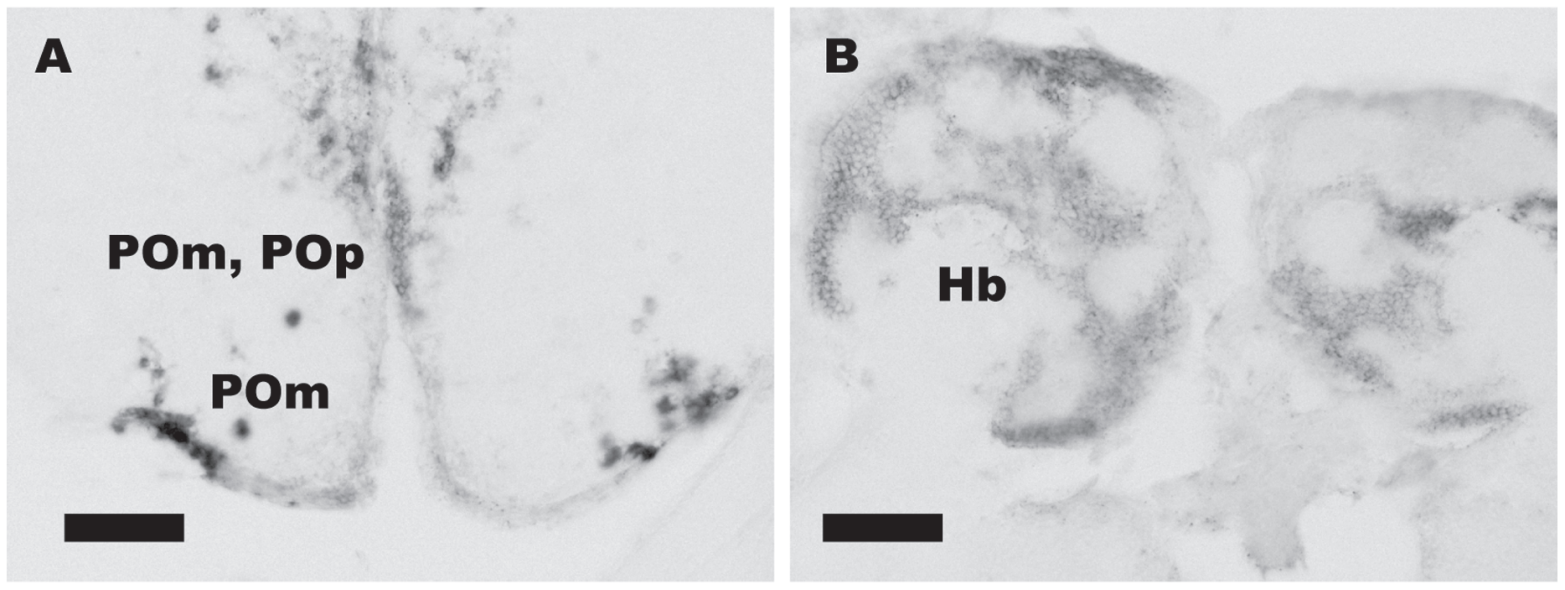
DIG-labelled *in situ* hybridization of *gpr54-1* shows localization of *gpr54-1* mRNA positive cells. *gpr54-1* mRNA positive neurons are localized in POp, POm (A), Vd/Vs/Vp (not shown), and habenula (Hb; B). Scale bars: 50 µm.

**Figure 3:**
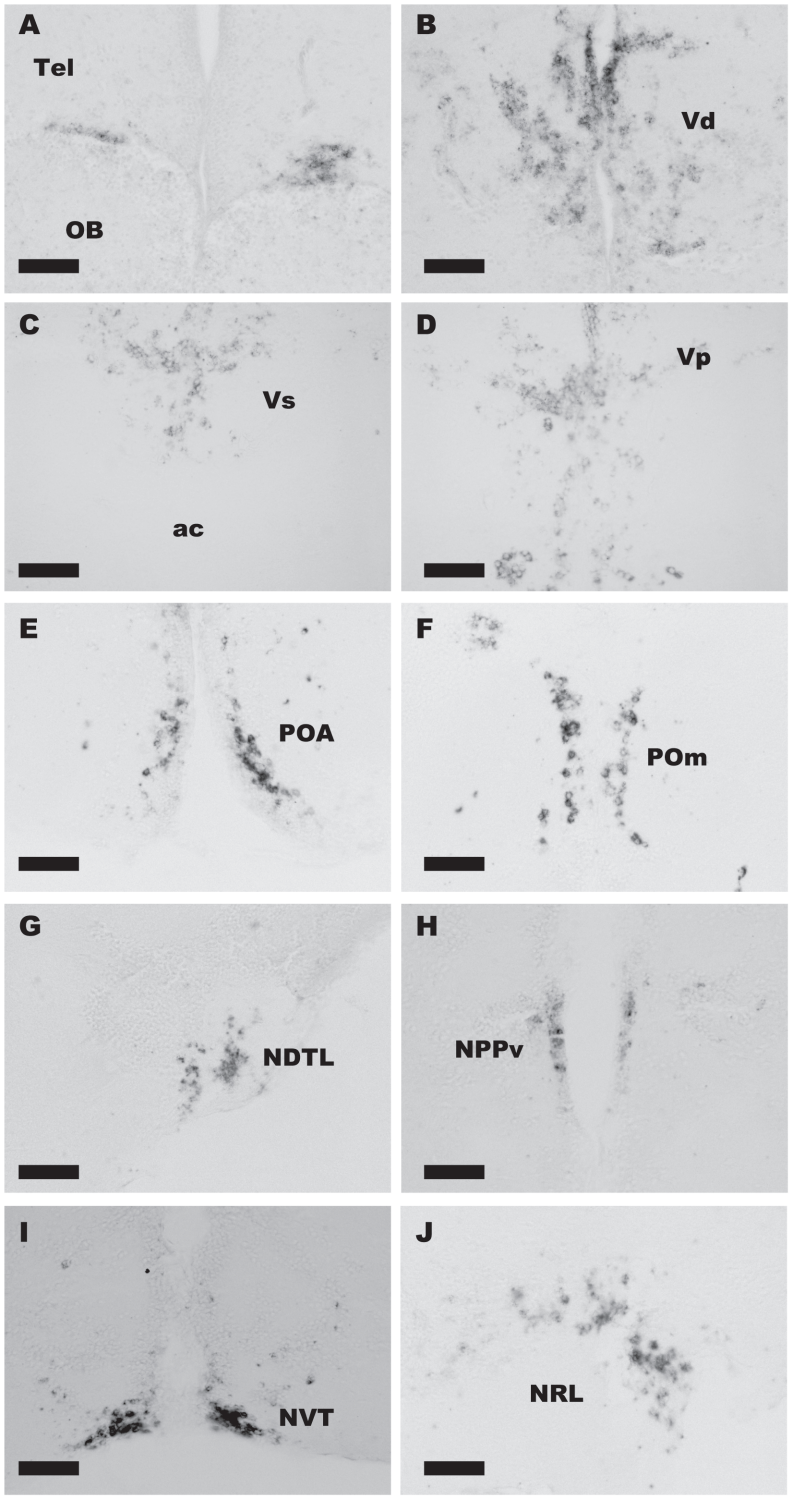
DIG-labelled ***in situ*** **hybridization of** ***gpr54-2*** **shows localization of gpr54-2 mRNA positive cells.** *gpr54-2* mRNA positive neurons are localized in the boundary between telencephalon (Tel) and olfactory bulb (OB; A), area ventralis telencephali pars dorsalis/supracommissuralis/posterior (Vd;B/Vs;C/Vp;D), area preoptica (POA; E), nucleus preopticus pars magnocellularis (POm; F), nucleus diffusus tori lateralis (NDTL; G), nucleus posterioris periventricularis (NPPv; H), nucleus ventralis tuberis (NVT; I), nucleus recessus lateralis (NRL; J), and corpus mammillare (CM, not shown). Scale bars: 50 µm.

*In situ* hybridization of two subtypes of kisspeptin receptors, *gpr54-1* and *gpr54-2*, was performed. Although the cDNA sequences of the two types of receptors are similar (61%), we could specifically label neurons that expressed *gpr54-1* (n = 3 for male, n = 8 for female) and neurons that expressed *gpr54-2* (n = 3 for male, n = 6 for female) as separate populations (Fig. 2, 3).

**Figure 4:**
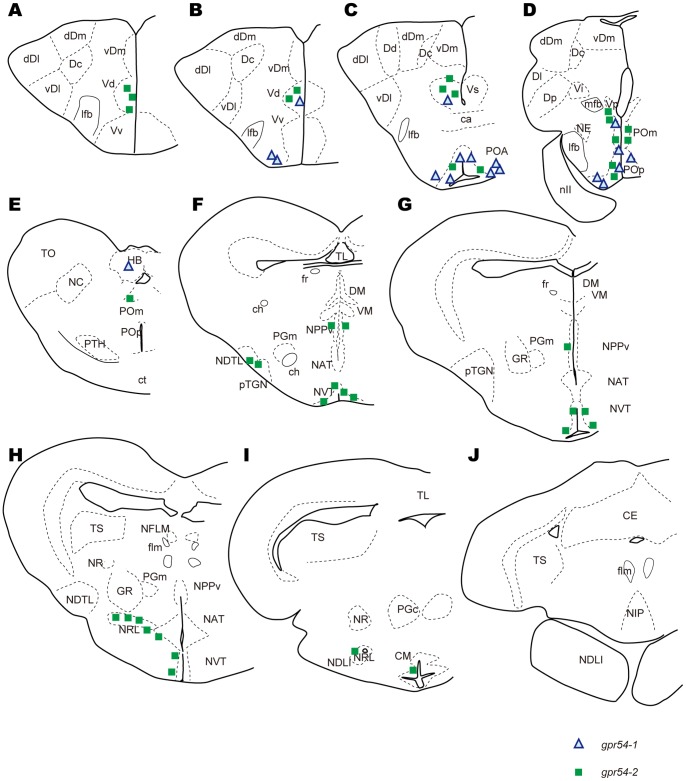
Schematic illustration of the distribution of kisspeptin receptors in medaka brain. *gpr54-1-* and *gpr54-2-*expressing neurons are mainly localized in the ventral telencephalon, preoptic area, and hypothalamus, suggesting their functions in homeostatic and behavioral regulations. Note that *gpr54-1* is also expressed in habenula but not in NIP, which is innervated by habenular neurons, suggesting the autocrine/paracrine regulation of habenular neurons by kisspeptins. Tel, telencephalon; TeO, optic tectum; ca, anterior commissure; Cb, cerebellum; Hy, hypothalamus; Hb, habenula; fr, fasciculus retroflexus; NIP, interpeduncular nucleus.

*In situ* hybridization of *gpr54-1* (Fig. 2) showed that the expression of *gpr54-1* mRNA is restricted in the preoptic area, nucleus preopticus pars magnocellularis (POm) and nucleus preopticus pars parvocellularis (POp) (Fig. 2A; Fig. 4D), area ventralis telencephali pars dorsalis/supracommissuralis/posterior (Vd/Vs/Vp), and habenula (Fig. 2B; Fig. 4E), where autocrine/paracrine regulation by kisspeptin is suggested in zebrafish [22], [23]. In the preoptic area, large cells in the ventralmost area expressed *gpr54-1*, while both large and small cells in the dorsomedial area expressed *gpr54-1.* On the other hand, *gpr54-2* mRNA showed broader distribution in the brain (Fig. 3), in the boundary between the telencephalon and the olfactory bulb (Fig. 3A), Vd/Vs/Vp (Fig. 3B–D; Fig. 4B–D), POA (Fig. 3E; Fig. 4C), POm (Fig. 3F; Fig. 4D), nucleus diffusus tori lateralis (NDTL; Fig. 3G; Fig. 4F), nucleus posterioris periventricularis (NPPv; Fig. 3H; Fig. 4F), NVT (Fig. 3I; Fig. 4F), NRL (Fig. 3J; Fig. 4H,I), and corpus mammillare (CM; Fig. 4I). There was no significant sex difference in the expression pattern of kisspeptin receptors throughout the brain. The distribution of kisspeptin receptor mRNA-expressing neurons in the medaka brain is schematically summarized in Fig. 4.

### Isotocin and Vasotocin Neurons Express Kisspeptin Receptors

**Figure 5:**
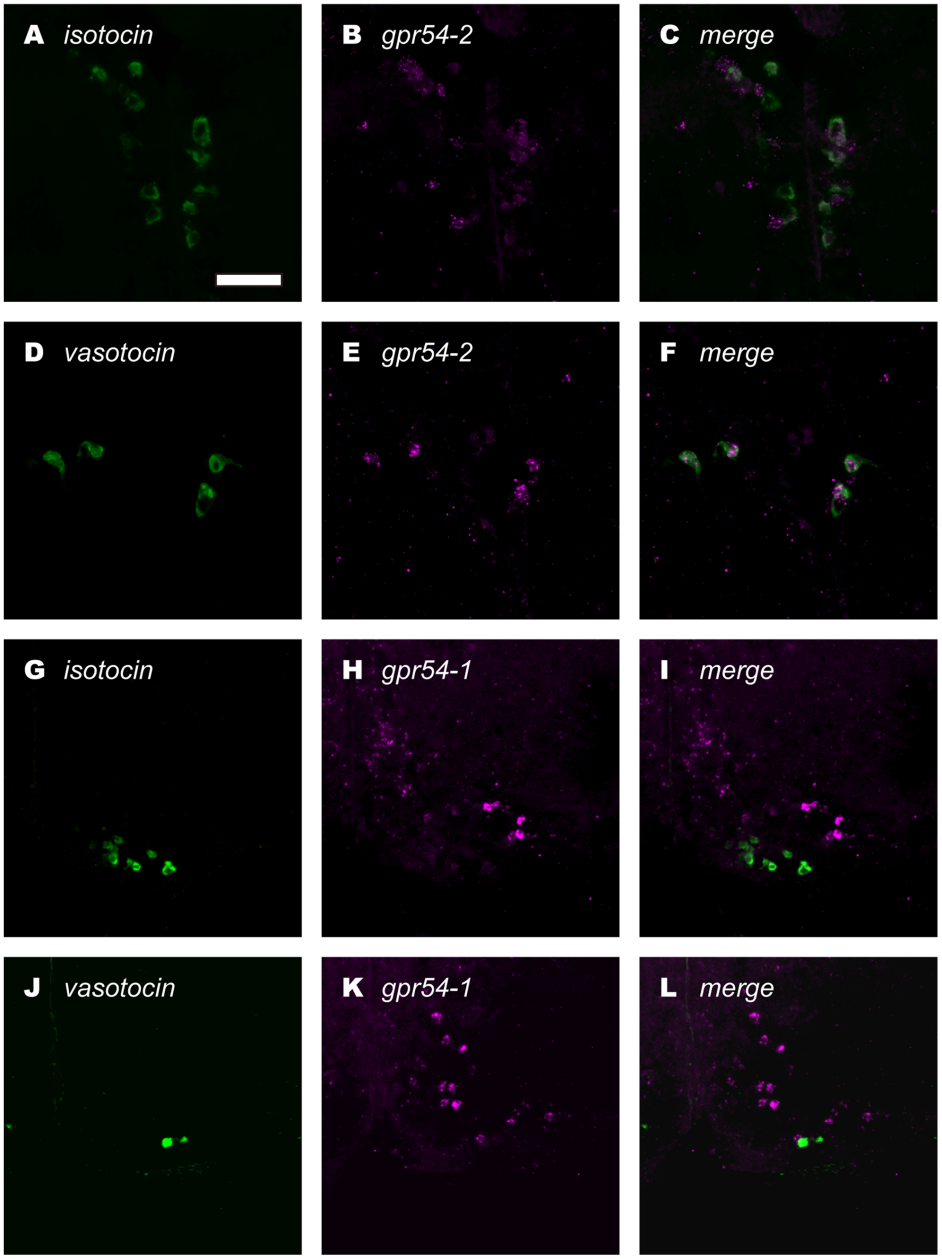
Dual fluorescence *in situ* hybridization showing that isotocin and vasotocin neurons express *gpr54-2*. Isotocin neurons (A; green) and vasotocin neurons (D; green) express *gpr54-2* (B, E; magenta). Merged photographs are shown in C and F. On the other hand, neurons that do not express isotocin or vasotocin (green) express *gpr54-1*(magenta) mRNA (G–I, and J–L, respectively). Scale bar: 50 µm.

We found large cells expressing *gpr54-2* mRNA in POm. Accordingly, we performed dual *in situ* hybridization to examine if they are isotocin and/or vasotocin neurons. We demonstrated that isotocin (Fig. 5A–C) as well as vasotocin neurons (Fig. 5D–F) express *gpr54-2*. In contrast, no isotocin (Fig. 5G–I) or vasotocin neurons (Fig. 5J–L) expressed *gpr54-1*. The percentage of co-localization was as follows. Vasotocin neurons; Female LD, 31±4% (n = 7), SD, 59±4% (n = 5), Male LD, 52±6% (n = 5), Male SD, 49±6% (n = 3): Isotocin neurons; Female LD, 19±1% (n = 3), SD, 18±1% (n = 2), Male LD, 15±3% (n = 4), SD17±2% (n = 3).

### Kisspeptin Receptors are Expressed in Proximity to GnRH1 Neurons

**Figure 6:**
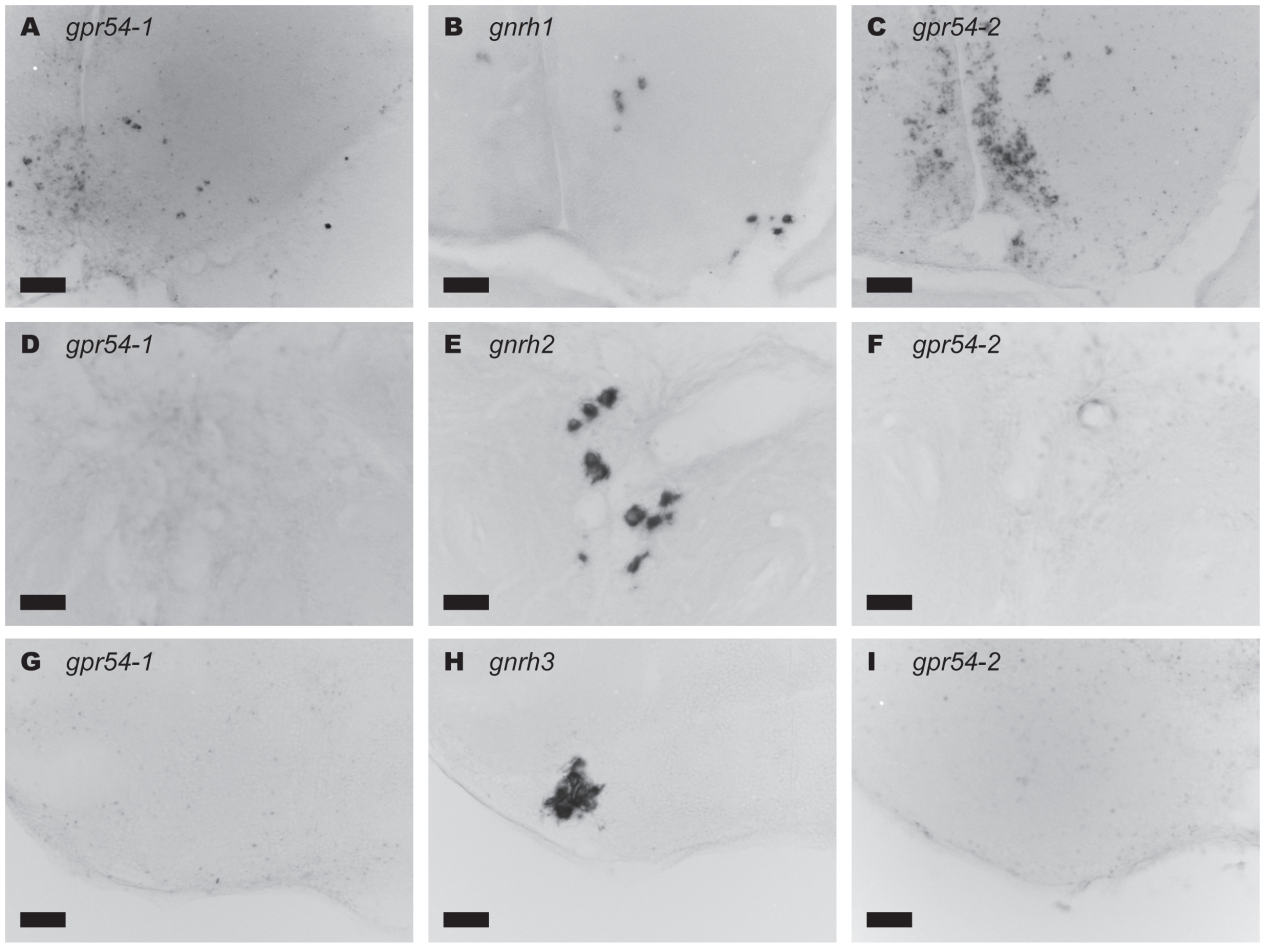
DIG-labelled *in situ* hybridization of the brain regions surrounding the three types of GnRH neurons (B: *gnrh1*, E: *gnrh2*, H: *gnrh3*), showing the presence or absence of expressions for the kisspeptin receptors, *gpr54-1* or *gpr54-2,* in these regions. The results for *gpr54-1* mRNA signals (A, D, G) and *gpr54-2* mRNA signals (C, F, I) show that the *gpr54-1*- and *gpr54-2*-expressing neurons are localized in the POA surrounding the GnRH1 neurons (A–C), but not in the midbrain tegmentum surrounding the GnRH2 neurons (D–F) or the ventral telencephalic area surrounding the GnRH3 neurons (G–I). Scale bars: 50 µm.

As described above, the *gpr54-1* and *gpr54-2* mRNA-expressing neurons were mainly localized in the ventral telencephalon, POA, habenula, and hypothalamus. Especially, we found that both *gpr54-1* and *gpr54-2* were expressed in POA surrounding the GnRH1 neurons (Fig. 6A–C; approximately 50 to 150 cells), but not in the regions surrounding the TEG-GnRH2 neurons (Fig. 6D–F) or TN-GnRH3 neurons (Fig. 6G–H).

**Figure 7:**
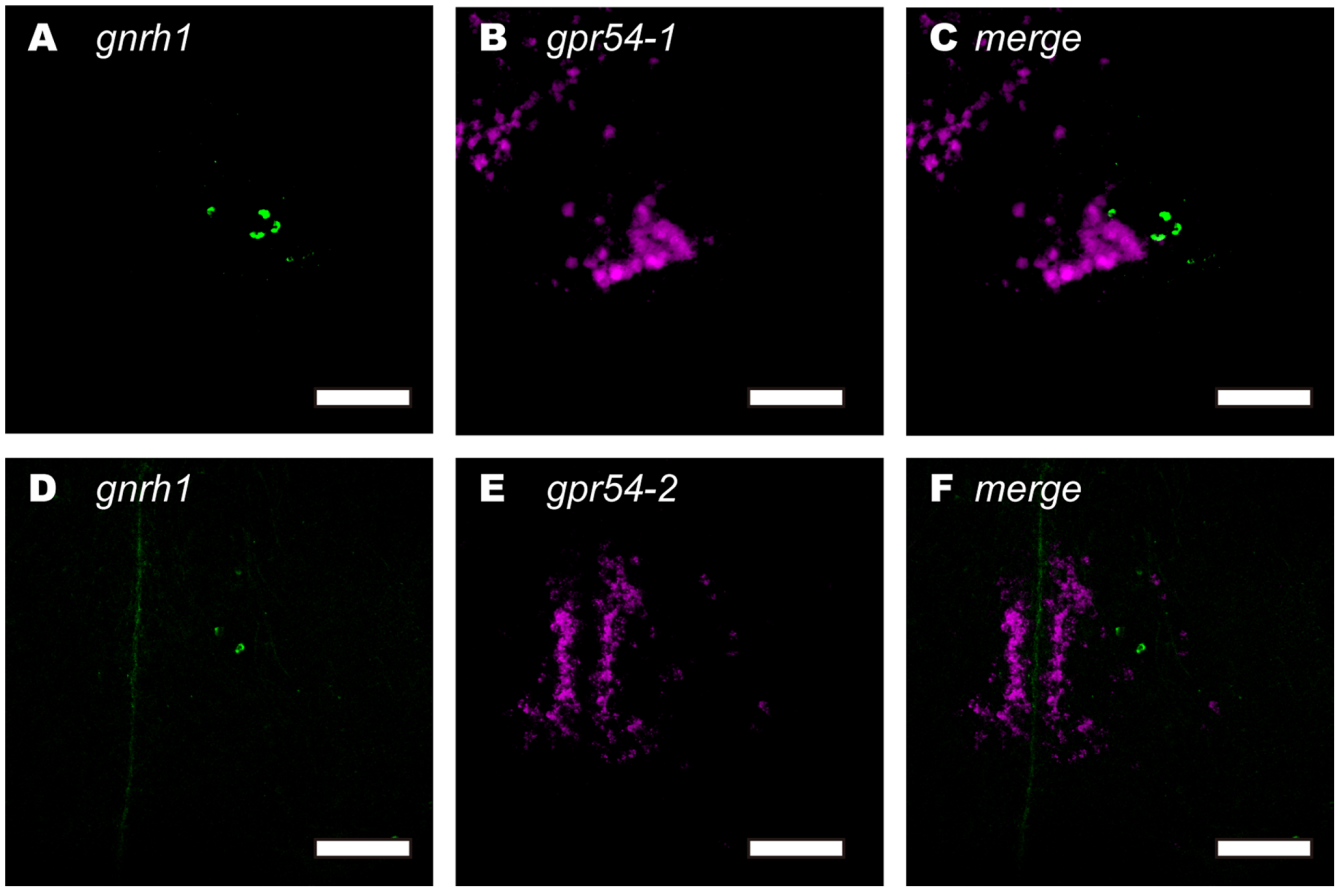
Dual fluorescence *in situ* hybridization showing that kisspeptin receptors (*gpr54-1* and *-2*) are highly expressed in the neurons (magenta) adjacent to the GnRH1 neurons (green) in the POA. *gpr54-1* mRNA (B) is expressed by neurons in proximity to the ventrolateral group of GnRH1 neurons in the POA (A; merged photo in C). *gpr54-2* mRNA (E) is expressed in neurons near the dorsomedial group of GnRH1 neurons in the POA (D; merged photo in F). Scale bar: 50 µm.

Since the *gpr54-1*- and *gpr54-2*-expressing neurons were localized in the proximity of the POA GnRH1 neurons, dual *in situ* hybridization combining either one of the two subtypes of kisspeptin receptor (*gpr54-1* or *gpr54-2*) mRNAs and *gnrh1* mRNA was performed (Fig. 7; n = 4 for male, n = 5 for female). However, at the single neuron level, neither *gpr54-1* nor *gpr54-2* mRNA was detected in the GnRH1 neurons themselves; they were abundantly expressed in the non-GnRH1 neurons surrounding the GnRH1 neurons (for *gpr54-1*, Fig. 7A–C; for *gpr54-2*, Fig. 7D–F). In contrast to the situation in the GnRH1 neurons, kisspeptin receptor was not expressed in proximity of neuromodulatory GnRH2 (Fig. 6D–F) or GnRH3 (Fig. 6G–I) neurons.

## Discussion

In the present study, we analyzed the distribution of kisspeptin receptors in a teleost medaka, and found the first evidence for kisspeptin’s direct regulation on magnocellular isotocin and vasotocin neurons in vertebrates. The kisspeptin receptors in the medaka brain showed characteristic distribution in that most of the neurons expressing *gpr54* mRNA were localized in the areas that have been suggested to be involved in the homeostatic regulations including reproduction and reproductive behaviors. We will mainly discuss below possible functions of kisspeptins and their receptors in non-mammalian vertebrates and its relevance to the vertebrate species in general.

### Gpr54-1 and Gpr54-2 are the Intrinsic Receptors for Both Kiss1 and Kiss2 in Medaka

Our luciferase assay has shown that both Kiss1 and Kiss2 activate both Gpr54-1 and Gpr54-2 signaling pathways. This result is consistent with the previous studies using zebrafish, African clawed frog, and goldfish [18], [20], [24]. In the present study, Kiss1 and Kiss2 activated Gpr54-2 SRE signaling to a similar extent (Fig. 1B), which has also been reported in zebrafish and goldfish [20], [24]. On the other hand, Kiss2 activated Gpr54-2 CRE signaling more potently than Kiss1 (Fig. 1D). Although the Gpr54-1-expressing COS-7 cells showed milder activation than Gpr54-2-expressing cells, they showed a clear dose dependent activation by Kiss1 and/or Kiss2 (Fig. 1A, C). Thus, it is concluded that both Gpr54-1 and Gpr54-2 are the intrinsic receptors for both Kiss1 and Kiss2 in medaka. These results lead us to perform the anatomical analysis of the distribution of both Gpr54-1 and Gpr54-2 in the medaka brain.

### Kisspeptin Receptors are Densely Expressed in POA and Hypothalamus

We demonstrated that the kisspeptin receptor genes, *gpr54-1* and *gpr54-2* are densely expressed in certain regions of ventral telencephalon, POA, and hypothalamus as well as habenula. Among those areas, *gpr54-1* was mainly expressed in ventral telencephalon, POA and habenula (Fig. 2), whereas *gpr54-2* was more widely expressed in the brain (Fig. 3). Previous study in a cichlid fish suggested that they lack *gpr54-1*, whereas *gpr54-2* mRNA is broadly expressed in the brain [25]. Moreover, in zebrafish, *gpr54-2* is expressed predominantly compared to *gpr54-1* [23]. Phylogenetically, in teleosts, many species lack *gpr54-1,* while *gpr54-2* is conserved throughout all the species analyzed to date. On the other hand, *gpr54-2* is lost in placental mammals [26], [27]. Taken together, our results and previous studies suggest that Gpr54-1 system may predominantly function in tetrapods, whereas Gpr54-2 system may do so in teleost brains.

As will be discussed in the next section, it is suggested that the kisspeptin receptors in POA are primarily involved in the control of release activities of hypophysiotropic hormones such as GnRH. In addition, previous brain lesion and stimulation studies in teleosts suggested that Vv, Vd/Vs/Vp, and POA are involved in the control of sexual behavior [28], [29]. In the hime salmon brain, electrical stimulation of these specific areas immediately evoked sexual behaviors, suggesting that these regions may function as an important part of the neural circuit for sexual behavior. In the goldfish, it has been shown that male sexual behaviors were severely impaired after bilateral lesions confined to the area ventralis telencephali pars supracommissuralis and/or posterior parts of the area ventralis telencephali pars ventralis (Vs-pVv) and the nucleus preopticus periventricularis (NPP) [29], which appear to overlap with the *gpr54-1*- and *gpr54-2*-expressing neurons in medaka.

### Evidence for the Direct Regulation of Kisspeptin Neurons on Isotocin and Vasotocin Neurons

We demonstrated clearly that vasotocin and isotocin neurons express *gpr54-2*. In mammals, some studies have shown that Kiss1 is involved in the control of release of oxytocin or vasopressin, although Kiss1’s site of action has not been clarified [30]–[32]. Some studies have also shown that vasotocin/vasopressin and isotocin/oxytocin neurons are involved in the control of social behaviors such as aggression and reproduction [33]–[36]. Given that *gpr54-2* can be activated by both Kiss1 and Kiss2, it is suggested that the isotocin and vasotocin neurons expressing *gpr54-2* are regulated by both Kiss1 and Kiss2 neurons. Previously, it has been reported by immunohistochemistry that medaka Kiss1 neurons project to POm [37]. Interestingly, medaka Kiss1 neurons change their *kiss1* expression levels according to the breeding states [16]. Although there is no report of Kiss2 neuronal projection in medaka, Kiss2 neurons in zebrafish are reported to project to the preoptic area [23]. Recently, POA Kiss2 neurons in goldfish, which belongs to the same Cyprinidae, were reported to show steroid dependent expression of *kiss2* mRNA [27], [38]. Thus, in teleosts, it is suggested that kisspeptin (Kiss1 and Kiss2) neurons project to isotocin/vasotocin neurons and show characteristic seasonal variations in their gene expression. Interestingly, in halfspotted goby the number and the size of arginine vasotocin and GnRH immunoreactive cells have been shown to be correlated with seasonal reproductivity [39]. Therefore, we propose that the Kiss1 and/or Kiss2 neurons may convey important information about the reproductive/gonadal states to the neural networks responsible for some reproductive and other behaviors that are regulated by vasotocin/isotocin neurons.

### Relationships between Kiss1 and Three GnRH Neuronal Systems

There are general agreements as to the concept that the POA GnRH1 neurons are hypophysiotropic, and the extrahypothalamic GnRH2 and GnRH3 neurons are non-hypophysiotropic and neuromodulatory in nature [15], [40], [41]. This principle appears to be widely conserved throughout vertebrate species including mammals [42]–[45].

In the present study, we demonstrated the anatomical relationships between the kisspeptin neurons and three different types of GnRH neurons for the first time in vertebrates by examining whether the kisspeptin receptors are localized to the GnRH neurons by using dual *in situ* hybridization.

We have clearly shown here that numerous neurons close to the GnRH1 neurons, but not the GnRH1 neurons themselves, expressed *gpr54-1* or *gpr54-2* (Figs. 6 and 7; also see below) in medaka. It was recently demonstrated in a cichlid fish (*Astatotilapia burtoni*) by *in situ* hybridization that kisspeptin receptor is expressed in GnRH3 neurons but not in GnRH2 neurons or GnRH1 neurons [25], which clearly shows that the adjacent neurons, but not GnRH1 neurons themselves, express kisspeptin receptors; the authors also stated that the receptor expression was much heavier in such non-GnRH1 neurons in the POA. This situation in teleosts is unique, because kisspeptin’s main function in mammals have been supposed to be the regulation of GnRH1 peptide release through Gpr54 on GnRH1 neurons (reviewed in [46]), and will be discussed in the next section. On the other hand, Kiss1 system does not appear to have such a pathway for the neuromodulatory GnRH2 neurons. There may be some species variations as to whether the GnRH3 neurons express kisspeptin receptors or not. Zhao and Wayne reported on changes in medaka GnRH3 firing frequency after the application of Kiss1 through some interneurons, which is consistent with the results of the present study, indicating that GnRH3 neurons in medaka do not express *gpr54* mRNA [47].

### Possible Pathway for the Indirect Kisspeptin Regulation of GnRH1 Neurons via Interneurons in POA

Our present dual *in situ* hybridization study clearly indicated that *gpr54*-expressing cells are located closely adjacent to the hypophysiotropic GnRH1 neurons but they are not the GnRH1 neurons themselves (Fig. 7). In mammals, kisspeptin is supposed to be an essential regulator of GnRH neuron activity. On the other hand, in teleosts, not many, but a few studies reported that kisspeptin up-regulates the expression of LHβ and FSHβ in zebrafish [48] and promotes LH secretion in goldfish [24] and sea bass [49], [50]. However, the direct action of kisspeptin on the pituitary was denied in goldfish [24]. We also examined possible direct action of Kiss1(10) on LHβ and FSHβ mRNA expression of the isolated medaka pituitary in culture. The Kiss1(10) peptide turned out to have no direct pituitary effect, while GnRH, as a positive control, potently enhanced their expressions in the same condition (data not shown). Thus, it may suggest that medaka Kiss1 indirectly regulates LH and FSH secretion probably via kisspeptin receptor-expressing interneurons in the POA in the vicinity of GnRH1 neurons, if the upregulation by kisspeptin of gonadotropin release (reported in some teleosts [24], [48], [50], [51]) is common to teleost species. It should be noted that in recent studies of mammalian kisspeptin neurons, Pielecka-Fortuna et al [6], [52] reported on the indirect kisspeptin regulation of GnRH neurons via interneurons, in addition to the direct regulation [7]–[9]. Therefore, such indirect kisspeptin regulation of GnRH neurons may be rather widely conserved in vertebrate species.

In the present study, we could not specify the identity of the neurons expressing kisspeptin receptors in the proximity of GnRH1 neurons from various technical reasons. The future identification of these neurons (the transmitter candidate and the anatomical and physiological nature) should be critical for understanding the mechanism of HPG axis regulation by kisspeptins.

In summary, by performing a systematic in situ hybridization analysis on the overall distribution of two types of kisspeptin receptors throughout the brain, we found evidence to show that kisspeptin neurons directly regulate isotocin and vasotocin neurons via Gpr54 for the first time in vertebrates. On the other hand, all three GnRH neuronal population were shown to lack the expression of kisspeptin receptors in medaka. Among them, however, neurons in proximity to GnRH1 neurons were shown to express *gpr54-1* or *gpr54-2* mRNA, leaving a possibility that kisspeptin indirectly regulates the GnRH1 system. Anyway, the expression of kisspeptin receptor in isotocin and vasotocin neurons suggests the following new functions of kisspeptin systems; the gonadal sex steroid-sensitive kisspeptin neurons (Kiss1 neurons in medaka) may alter reproduction-related behaviors by affecting isotocin and vasotocin neurons in accordance with the breeding states. Because isotocin and vasotocin neurons are homologous to mammalian oxytocin and vasopressin neurons, similar regulation may also exist in mammalian oxytocin and vasopressin neurons. Thus, the elucidation of regulatory mechanisms of teleost kisspeptin neurons on vasotocin and isotocin neurons may open the new era of research of kisspeptin neurons’ physiological functions in vertebrates.

## References

[1] de Roux N, Genin E, Carel JC, Matsuda F, Chaussain JL, et al (2003) Hypogonadotropic hypogonadism due to loss of function of the KiSS1-derived peptide receptor GPR54. Proc Natl Acad Sci U S A 100: 10972–10976. 12944565 10.1073/pnas.1834399100 PMC196911

[2] Funes S, Hedrick JA, Vassileva G, Markowitz L, Abbondanzo S, et al (2003) The KiSS-1 receptor GPR54 is essential for the development of the murine reproductive system. Biochem Biophys Res Commun 312: 1357–1363. 14652023 10.1016/j.bbrc.2003.11.066

[3] Seminara SB, Messager S, Chatzidaki EE, Thresher RR, Acierno JS, et al (2003) The GPR54 gene as a regulator of puberty. N Engl J Med 349: 1614–1627. 14573733 10.1056/NEJMoa035322

[4] d’Anglemont de Tassigny X, Fagg LA, Dixon JP, Day K, Leitch HG, et al (2007) Hypogonadotropic hypogonadism in mice lacking a functional Kiss1 gene. Proc Natl Acad Sci U S A 104: 10714–10719. 17563351 10.1073/pnas.0704114104 PMC1965578

[5] Lapatto R, Pallais JC, Zhang D, Chan YM, Mahan A, et al (2007) Kiss1−/− mice exhibit more variable hypogonadism than Gpr54−/− mice. Endocrinology 148: 4927–4936. 17595229 10.1210/en.2007-0078

[6] Pielecka-Fortuna J, Chu Z, Moenter SM (2008) Kisspeptin acts directly and indirectly to increase gonadotropin-releasing hormone neuron activity and its effects are modulated by estradiol. Endocrinology 149: 1979–1986. 18162521 10.1210/en.2007-1365 PMC2276721

[7] Liu X, Lee K, Herbison AE (2008) Kisspeptin excites gonadotropin-releasing hormone neurons through a phospholipase C/calcium-dependent pathway regulating multiple ion channels. Endocrinology 149: 4605–4614. 18483150 10.1210/en.2008-0321 PMC6116891

[8] Zhang C, Roepke TA, Kelly MJ, Ronnekleiv OK (2008) Kisspeptin depolarizes gonadotropin-releasing hormone neurons through activation of TRPC-like cationic channels. J Neurosci 28: 4423–4434. 18434521 10.1523/JNEUROSCI.5352-07.2008 PMC6670958

[9] Han SK, Gottsch ML, Lee KJ, Popa SM, Smith JT, et al (2005) Activation of gonadotropin-releasing hormone neurons by kisspeptin as a neuroendocrine switch for the onset of puberty. J Neurosci 25: 11349–11356. 16339030 10.1523/JNEUROSCI.3328-05.2005 PMC6725899

[10] Dumalska I, Wu M, Morozova E, Liu R, van den Pol A, et al (2008) Excitatory effects of the puberty-initiating peptide kisspeptin and group I metabotropic glutamate receptor agonists differentiate two distinct subpopulations of gonadotropin-releasing hormone neurons. J Neurosci 28: 8003–8013. 18685025 10.1523/JNEUROSCI.1225-08.2008 PMC2597556

[11] Goodson JL, Thompson RR (2010) Nonapeptide mechanisms of social cognition, behavior and species-specific social systems. Curr Opin Neurobiol 20: 784–794. 20850965 10.1016/j.conb.2010.08.020

[12] Meyer-Lindenberg A, Domes G, Kirsch P, Heinrichs M (2011) Oxytocin and vasopressin in the human brain: social neuropeptides for translational medicine. Nat Rev Neurosci 12: 524–538. 21852800 10.1038/nrn3044

[13] Godwin J, Thompson R (2012) Nonapeptides and social behavior in fishes. Horm Behav 61: 230–238. 22285647 10.1016/j.yhbeh.2011.12.016

[14] Okubo K, Nagahama Y (2008) Structural and functional evolution of gonadotropin-releasing hormone in vertebrates. Acta Physiol (Oxf) 193: 3–15. 18284378 10.1111/j.1748-1716.2008.01832.x

[15] Oka Y (2009) Three types of GnRH neurones and steroid-sensitive sexually dimorphic kisspeptin neurones in teleosts. J Neuroendocrinol 21: 334–338. 19210296 10.1111/j.1365-2826.2009.01850.x

[16] Kanda S, Akazome Y, Matsunaga T, Yamamoto N, Yamada S, et al (2008) Identification of KiSS-1 Product Kisspeptin and Steroid-Sensitive Sexually Dimorphic Kisspeptin Neurons in Medaka (Oryzias latipes). Endocrinology 149: 2467–2476. 18202129 10.1210/en.2007-1503

[17] Kawabata Y, Hiraki T, Takeuchi A, Okubo K (2012) Sex differences in the expression of vasotocin/isotocin, gonadotropin-releasing hormone, and tyrosine and tryptophan hydroxylase family genes in the medaka brain. Neuroscience. 10.1016/j.neuroscience.2012.05.021 22609934

[18] Biran J, Ben-Dor S, Levavi-Sivan B (2008) Molecular identification and functional characterization of the kisspeptin/kisspeptin receptor system in lower vertebrates. Biol Reprod 79: 776–786. 18509165 10.1095/biolreprod.107.066266

[19] Akazome Y, Kanda S, Okubo K, Oka Y (2010) Functional and evolutionary insights into vertebrate kisspeptin systems from studies of fish brain. Journal of Fish Biology 76: 161–182. 20738704 10.1111/j.1095-8649.2009.02496.x

[20] Lee YR, Tsunekawa K, Moon MJ, Um HN, Hwang JI, et al (2009) Molecular evolution of multiple forms of kisspeptins and GPR54 receptors in vertebrates. Endocrinology 150: 2837–2846. 19164475 10.1210/en.2008-1679

[21] Kanda S, Okubo K, Oka Y (2011) Differential regulation of the luteinizing hormone genes in teleosts and tetrapods due to their distinct genomic environments - Insights into gonadotropin beta subunit evolution. Gen Comp Endocrinol 173: 253–258. 21663743 10.1016/j.ygcen.2011.05.015

[22] Ogawa S, Ng KW, Ramadasan PN, Nathan FM, Parhar IS (2012) Habenular kiss1 neurons modulate the serotonergic system in the brain of zebrafish. Endocrinology 153: 2398–2407. 22454151 10.1210/en.2012-1062

[23] Servili A, Le Page Y, Leprince J, Caraty A, Escobar S, et al (2011) Organization of two independent kisspeptin systems derived from evolutionary-ancient kiss genes in the brain of zebrafish. Endocrinology 152: 1527–1540. 21325050 10.1210/en.2010-0948

[24] Li S, Zhang Y, Liu Y, Huang X, Huang W, et al (2009) Structural and functional multiplicity of the kisspeptin/GPR54 system in goldfish (Carassius auratus). J Endocrinol 201: 407–418. 19304758 10.1677/JOE-09-0016

[25] Grone BP, Maruska KP, Korzan WJ, Fernald RD (2010) Social status regulates kisspeptin receptor mRNA in the brain of Astatotilapia burtoni. Gen Comp Endocrinol 169: 98–107. 20688063 10.1016/j.ygcen.2010.07.018 PMC2951738

[26] Kim DK, Cho EB, Moon MJ, Park S, Hwang JI, et al (2012) Molecular Coevolution of Neuropeptides Gonadotropin-Releasing Hormone and Kisspeptin with their Cognate G Protein-Coupled Receptors. Front Neurosci 6: 3. 22291614 10.3389/fnins.2012.00003 PMC3265131

[27] Kanda S, Oka Y (2012) Evolutionary insights into the steroid sensitive *kiss1* and *kiss2* neurons in the vertebrate brain. Frontiers in Endocrinology 3: doi: 10.3389/fendo.2012.00028. 10.3389/fendo.2012.00028 PMC3356069 22654859

[28] Satou M, Oka Y, Kusunoki M, Matsushima T, Kato M, et al (1984) Telencephalic and preoptic areas integrate sexual behavior in hime salmon (landlocked red salmon, Oncorhynchus nerka): results of electrical brain stimulation experiments. Physiol Behav 33: 441–447. 6393162 10.1016/0031-9384(84)90167-7

[29] Koyama Y, Satou M, Oka Y, Ueda K (1984) Involvement of the telencephalic hemispheres and the preoptic area in sexual behavior of the male goldfish, Carassius auratus: a brain-lesion study. Behav Neural Biol 40: 70–86. 6610412 10.1016/s0163-1047(84)90182-1

[30] Kotani M, Detheux M, Vandenbogaerde A, Communi D, Vanderwinden JM, et al (2001) The metastasis suppressor gene KiSS-1 encodes kisspeptins, the natural ligands of the orphan G protein-coupled receptor GPR54. J Biol Chem 276: 34631–34636. 11457843 10.1074/jbc.M104847200

[31] Ten SC, Gu SY, Niu YF, An XF, Yan M, et al (2010) Central administration of kisspeptin-10 inhibits water and sodium excretion of anesthetized male rats and the involvement of arginine vasopressin. Endocr Res 35: 128–136. 20712435 10.3109/07435801003769995

[32] Scott V, Brown CH (2011) Kisspeptin activation of supraoptic nucleus neurons in vivo. Endocrinology 152: 3862–3870. 21810945 10.1210/en.2011-1181

[33] Oldfield RG, Hofmann HA (2011) Neuropeptide regulation of social behavior in a monogamous cichlid fish. Physiol Behav 102: 296–303. 21112347 10.1016/j.physbeh.2010.11.022

[34] Winslow JT, Hastings N, Carter CS, Harbaugh CR, Insel TR (1993) A role for central vasopressin in pair bonding in monogamous prairie voles. Nature 365: 545–548. 8413608 10.1038/365545a0

[35] Greenwood AK, Wark AR, Fernald RD, Hofmann HA (2008) Expression of arginine vasotocin in distinct preoptic regions is associated with dominant and subordinate behaviour in an African cichlid fish. Proc Biol Sci 275: 2393–2402. 18628117 10.1098/rspb.2008.0622 PMC2603226

[36] Goodson JL, Bass AH (2001) Social behavior functions and related anatomical characteristics of vasotocin/vasopressin systems in vertebrates. Brain Res Brain Res Rev 35: 246–265. 11423156 10.1016/s0165-0173(01)00043-1

[37] Kanda S, Akazome Y, Okubo K, Okamura H, Oka Y (2009) Kisspeptin neurons act closely but indirectly on GnRH 1 neurons via local interneurons but not on GnRH 2 or 3 neurons in medaka; Chicago, IL.

[38] Kanda S, Karigo T, Oka Y (2012) Steroid sensitive kiss2 neurones in the goldfish: evolutionary insights into the duplicate kisspeptin gene-expressing neurones. J Neuroendocrinol 24: 897–906. 22340198 10.1111/j.1365-2826.2012.02296.x

[39] Maruska KP, Mizobe MH, Tricas TC (2007) Sex and seasonal co-variation of arginine vasotocin (AVT) and gonadotropin-releasing hormone (GnRH) neurons in the brain of the halfspotted goby. Comp Biochem Physiol A Mol Integr Physiol 147: 129–144. 17276115 10.1016/j.cbpa.2006.12.019

[40] Kanda S, Nishikawa K, Karigo T, Okubo K, Isomae S, et al (2010) Regular pacemaker activity characterizes gonadotropin-releasing hormone 2 neurons recorded from green fluorescent protein-transgenic medaka. Endocrinology 151: 695–701. 20032054 10.1210/en.2009-0842

[41] Karigo T, Kanda S, Takahashi A, Abe H, Okubo K, et al (2012) Time-of-Day-Dependent Changes in GnRH1 Neuronal Activities and Gonadotropin mRNA Expression in a Daily Spawning Fish, Medaka. Endocrinology 153: 3394–3404. 22544888 10.1210/en.2011-2022

[42] Wirsig CR, Leonard CM (1987) Terminal nerve damage impairs the mating behavior of the male hamster. Brain Res 417: 293–303. 3308003 10.1016/0006-8993(87)90454-9

[43] Kauffman AS (2004) Emerging functions of gonadotropin-releasing hormone II in mammalian physiology and behaviour. J Neuroendocrinol 16: 794–806. 15344918 10.1111/j.1365-2826.2004.01229.x

[44] Kauffman AS, Rissman EF (2004) A critical role for the evolutionarily conserved gonadotropin-releasing hormone II: mediation of energy status and female sexual behavior. Endocrinology 145: 3639–3646. 15105381 10.1210/en.2004-0148

[45] Kauffman AS, Bojkowska K, Wills A, Rissman EF (2006) Gonadotropin-releasing hormone-II messenger ribonucleic acid and protein content in the mammalian brain are modulated by food intake. Endocrinology 147: 5069–5077. 16873537 10.1210/en.2006-0615

[46] Oakley AE, Clifton DK, Steiner RA (2009) Kisspeptin signaling in the brain. Endocr Rev 30: 713–743. 19770291 10.1210/er.2009-0005 PMC2761114

[47] Zhao Y, Wayne NL (2012) Effects of Kisspeptin1 on Electrical Activity of an Extrahypothalamic Population of Gonadotropin-Releasing Hormone Neurons in Medaka (Oryzias latipes). PLoS One 7: e37909. 22649563 10.1371/journal.pone.0037909 PMC3359290

[48] Kitahashi T, Ogawa S, Parhar IS (2009) Cloning and Expression of kiss2 in the Zebrafish and Medaka. Endocrinology 150: 821–831. 18927220 10.1210/en.2008-0940

[49] Felip A, Zanuy S, Pineda R, Pinilla L, Carrillo M, et al.. (2008) Evidence for two distinct KiSS genes in non-placental vertebrates that encode kisspeptins with different gonadotropin-releasing activities in fish and mammals. Mol Cell Endocrinol. 10.1016/j.mce.2008.11.017 19084576

[50] Zmora N, Stubblefield J, Zulperi Z, Biran J, Levavi-Sivan B, et al.. (2012) Differential and Gonad Stage-Dependent Roles of Kisspeptin1 and Kisspeptin2 in Reproduction in the Modern Teleosts, Morone Species. Biol Reprod. 10.1095/biolreprod.111.097667 22423047

[51] Felip A, Zanuy S, Pineda R, Pinilla L, Carrillo M, et al (2009) Evidence for two distinct KiSS genes in non-placental vertebrates that encode kisspeptins with different gonadotropin-releasing activities in fish and mammals. Mol Cell Endocrinol 312: 61–71. 19084576 10.1016/j.mce.2008.11.017

[52] Pielecka-Fortuna J, Moenter SM (2010) Kisspeptin increases gamma-aminobutyric acidergic and glutamatergic transmission directly to gonadotropin-releasing hormone neurons in an estradiol-dependent manner. Endocrinology 151: 291–300. 19880809 10.1210/en.2009-0692 PMC2803153

